# The Relative Performance of a Benchtop Scanning Monochromator and Handheld Fourier Transform Near-Infrared Reflectance Spectrometer in Predicting Forage Nutritive Value

**DOI:** 10.3390/s22020658

**Published:** 2022-01-15

**Authors:** Matthew F. Digman, Jerry H. Cherney, Debbie J. R. Cherney

**Affiliations:** 1 Department of Biological Systems Engineering, University of Wisconsin-Madison, Madison, WI 53706, USA; 2 Section of Soil and Crop Sciences, School of Integrative Plant Science, Cornell University, Ithaca, NY 14853, USA; jhc5@cornell.edu; 3 Department of Animal Science, Cornell University, Ithaca, NY 14853, USA; djc6@cornell.edu

**Keywords:** NIRS, FT-NIRS, forage quality, Michelson interferometer

## Abstract

Advanced manufacturing techniques have enabled low-cost, on-chip spectrometers. Little research exists, however, on their performance relative to the state of technology systems. The present study compares the utility of a benchtop FOSS NIRSystems 6500 (FOSS) to a handheld NeoSpectra-Scanner (NEO) to develop models that predict the composition of dried and ground grass, and alfalfa forages. Mixed-species prediction models were developed for several forage constituents, and performance was assessed using an independent dataset. Prediction models developed with spectra from the FOSS instrument had a standard error of prediction (SEP, % DM) of 1.4, 1.8, 3.3, 1.0, 0.42, and 1.3, for neutral detergent fiber (NDF), true in vitro digestibility (IVTD), neutral detergent fiber digestibility (NDFD), acid detergent fiber (ADF), acid detergent lignin (ADL), and crude protein (CP), respectively. The R^2^P for these models ranged from 0.90 to 0.97. Models developed with the NEO resulted in an average increase in SEP of 0.14 and an average decrease in R^2^P of 0.002.

## 1. Introduction

Forages are the primary ingredient in ruminant nutrition. The nutrient composition of fed rations differs from formulated rations due to variation in dry matter content and nutrient composition of forages [1]. Without continuous monitoring and adjustment, this variation can result in nutrient deficiencies. However, this risk is often mitigated by formulating rations with added nutrients, resulting in increased feed costs and excess nutrient loss to the environment [2].

Near-infrared reflectance spectroscopy (NIRS) has been used to evaluate foraged nutritive value for nearly forty years [3]. NIRS reduces the cost of forage analysis by reducing the need for chemical and in vitro digestibility assays, that are labor-intensive and expensive. Utilizing these data, livestock producers adjust the composition of the diet to maintain animal health and productivity. Although NIRS instrument technology has evolved, the scanning monochromator, specifically the FOSS NIRSystem 6500, remains one of the most common laboratory instruments in the feed and forage industry. This system is only recently being displaced by diode array instrument technology, as FOSS discontinues support by the end of this year [4].

Diode array spectrometer technology has enabled new applications of NIRS, including on-harvester systems [5,6,7] and recent handheld devices [8,9,10,11,12]. In addition to diode array instrument technology, advanced manufacturing techniques have led to the advent of new spectrometer designs that use micro-electro-mechanical systems (MEMS) technology (for review, see Bec et al. [13]). These instruments promise to reduce cost and size while further improving the robustness of NIR spectrometers.

One such device utilizes a digital micromirror device to focus diffracted light onto a single InGaAs photodetector. Using a spectrometer employing this technology, Acosta et al. [9] and Berzaghi et al. [10] demonstrated the utility to predict forage nutritive value in dried and ground samples, relative to the FOSS NIRSystem 6500. Since that time, an additional MEMS device has reached commercial availability. Marketed under the tradename NeoSpectra (Siware Systems Inc., Cairo, Egypt), this instrument utilizes a semiconductor manufacturing technique known as photolithography, to implement a Michelson interferometer onto a single MEMS chip, as a component of a Fourier transform near-infrared spectrometer (FT-NIR) [13]. The chip has been packaged into both an OEM module (NeoSpectra-Micro, Si-Ware Systems Inc., Cairo, Egypt) and a handheld device (NeoSpectra-Scanner, Si-Ware Systems Inc., Cairo, Egypt) at the time of writing. The handheld device is the subject of this study.

The objective of this study was to develop models to predict forage nutritive value (fiber, crude protein) using the NeoSpectra-Scanner and FOSS NIRSystems 6500 instruments, with dried and ground fresh alfalfa and grass samples, and to determine the relative performance on an independent set of samples.

## 2. Materials and Methods

### 2.1. Sample Database

Alfalfa (n = 104) and grass (n = 180) samples were compiled from alfalfa and grass experiments conducted in 2017, 2018, and 2020, at field sites in central and northern New York, USA. Ten alfalfa cultivars and 20 grass cultivars from two grass species: tall fescue (*Lolium arundinaceum* (Schreb.) S.J. Darbyshire) and meadow fescue (*Schedonorus pratensis* (Huds.) P. Beauv.; syn. *Festuca pratensis* Huds.; syn. *Lolium pratense* (Huds.) Darbysh.). Alfalfa and grass were either pure or mixed stands, with samples collected from early spring to late fall. Forage samples were hand-harvested at a 10 cm stubble height with a battery-powered clipper, from an approximately 0.25 m^2^ area that varied depending on the growth stage. All samples were dried in forced-air ovens to a constant weight at 60 °C, and ground in a Wiley mill (Thomas Scientific, Swedesboro, NJ, USA) to pass through a 1 mm sieve, and placed in labeled sealable plastic cups.

### 2.2. Laboratory Reference Methods

Samples were analyzed using wet chemistry procedures described in Valentine et al. [14], using sodium sulfite in the neutral detergent solution. Forages were weighed into ANKOM F57 filter bags (ANKOM Technology, Macedon, NY, USA) for NDF, ADF, ADL, and 48 h in vitro digestibility (IVTD) analyses. Filter bags were removed briefly from jars at the start and end of the second day for all in vitro digestion runs, and gas buildup was gently expressed while jars were being purged with CO_2_. Neutral detergent fiber digestibility (NDFD) was calculated as the proportion of the total fiber digested, reported on an NDF basis.

Nitrogen was determined using a combustion process (LECO CN628 analyzer, DairyOne, Ithaca, NY, USA), and crude protein (CP) was calculated as N × 6.25 [15]. All analyses were conducted in duplicate, except for nitrogen, which was determined in duplicate on a subset of samples, to calculate a standard error of the laboratory for CP. The standard error of the laboratory (SEL) for these analyses has been reported previously [8].

### 2.3. Instruments

The (FOSS) NIRSystem 6500 (FOSS, Hillerod, Denmark) is a scanning monochromator spectrometer with a wavelength range from 1100 to 2498 nm and reports data at 2 nm resolution. Dried and ground forage samples were loaded into the instrument equipped with a sample transport module, using a transport quarter cup with a quartz window.

**Figure 1:**
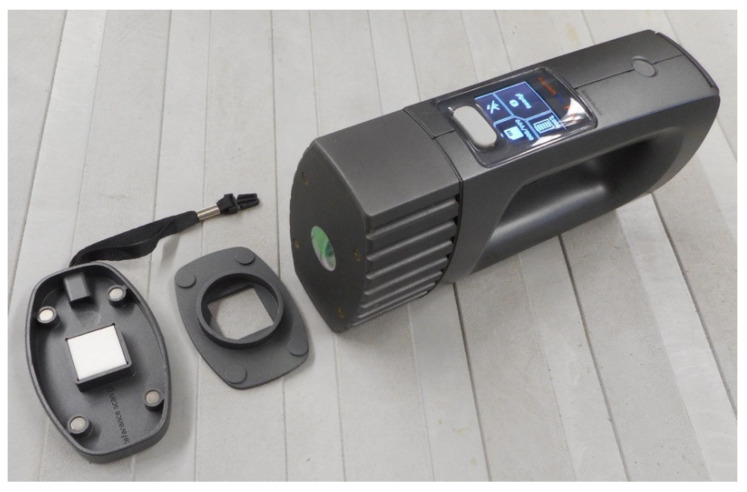
NeoSpec-Scanner, sample holder, and white reference.

The (NEO) NeoSpectra-Scanner (Si-Ware Systems Inc., Cairo, Egypt) integrates the following into a handheld device: battery power, a light source, light collection optics, a monolithic Michelson interferometer, an uncooled InGaAs photodetector, system control, data processing, and Bluetooth connectivity electronics (Figure 1). Data were collected with the NeoSpectra Scan software V1.0 on an Android tablet. The device has a 10 mm collection window, and the software reports spectra from 1350 to 2550 nm at a variable step between 2.5 and 8.8 nm, and a wavelength resolution of 16 nm. Duplicate scans of the forage samples were collected with the device and averaged.

The two instruments had different wavelength ranges. The FOSS instrument reported a reflectance spectrum from 1100 to 2498 nm and the NEO from 1350 to 2550 nm. Based on previous research, it is well established that the 1100–1350 nm region has limited utility in predicting forage nutritive value [16]. Therefore, the FOSS instrument and NEO instruments were trimmed to an overlapping range of 1350 to 2498 nm.

### 2.4. Model Development

Partial least square (PLS) regression was used to explore the relationship between response (forage constituent laboratory values) and predictor variables (spectra). PLSR was implemented in Mathematica (Version 12.1, Wolfram Research, Inc., Champaign, IL, USA) using a non-linear iterative PLS algorithm [17].

Prediction models were developed for each instrument (NEO, FOSS), forage species (fresh alfalfa, grass), and forage constituent (NDF, IVTD, NDFD, ADF, ADL, CP). A mixed forage species model was also considered. In all calibration models, spectra from repeated scans were averaged, converted to absorbance (log R^−^^1^) and mean-centered. Additional spectral preprocessing techniques explored included none, standard normal variate and detrend, Savitzky–Golay smoothing, first derivative, and second derivative. For the Savitzky–Golay smoothing and derivative treatments, a sensitivity analysis was performed for the derivative order: none (smoothing), first or second, window width: 9–27 variables, and the polynomial order: two or three.

The number of latent variables was established by meeting the threshold of explaining 95% of the variance in X and that at least 10 data points supported each LV. This approach was used to generate an automated and systematic comparison of the LVs for each instrument. However, the result was compared to the plot of the variance explained for each LV for both calibration and cross-validation sets, to ensure this criterion did not overfit the model. Cross-validation utilized the venetian blinds method with five splits and a blind thickness of one.

The impact of spectral preprocessing on the number of latent variables selected, standard error of calibration (SEC), standard error of five-fold cross-validation (SECV), and coefficient of determination (R^2^C(V)) was considered before selecting the optimal spectral transformation. The SEC(V) was determined as

(1) $$\mathrm{SEC}\left(V\right)=\sqrt{\frac{\sum_{i=1}^{N}{\left(L_{i}-P_{i}\right)}^{2}}{N-1-\mathrm{LV}}}$$

where L = laboratory reference, P = NIRS predicted value, N = number of samples, and LV is the number of latent variables in the model.

Ultimately, the performance of the models was assessed using a validation set. Before the model development, the odd harvest dates were segregated. Prediction of the validation dataset established model performance. These results were described by the coefficient of determination (R^2^P), root mean square error of prediction

(2) $$\mathrm{RMSEP}=\sqrt{\frac{\sum_{i=1}^{N}{\left(L_{i}-P_{i}\right)}^{2}}{N-1}}$$

and the RMSEP corrected for the bias or standard error of prediction

(3) $$\mathrm{SEP}=\sqrt{\frac{\sum_{i=1}^{N}{\left(L_{i}-P_{i}\right)}^{2}-\frac{{\left(\sum_{i=1}^{N}\left(L_{i}-P_{i}\right)\right)}^{2}}{N}}{N-1}}$$

## 3. Results and Discussion

**Figure 2:**
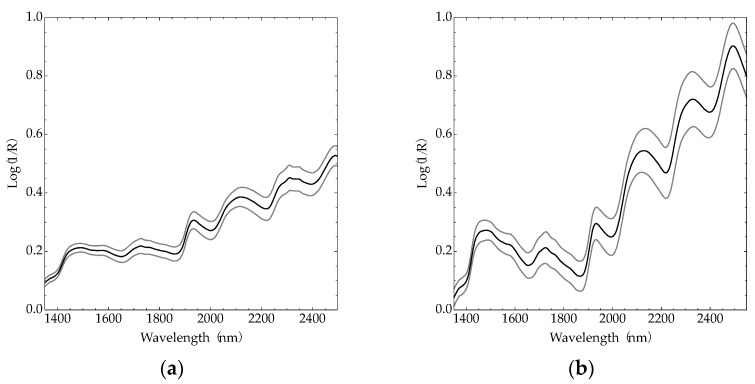
(**a**) Mean and standard deviation of spectra collected from 384 dried and ground forage samples collected with the FOSS NIRSystems 6500; (**b**) Mean and standard deviation of spectra collected from 384 dried and ground forage samples collected with the NeoSpec-Scanner.

The average and one standard deviation of the spectra, collected from all forage samples with the FOSS and NEO spectrometers, are presented in Figure 2. Although the average spectra between the two instruments appear dissimilar, the spectra were highly correlated. To compare the spectra between the instruments, linear models were fit to the absorbance reported by each spectrometer at each wavelength with an average coefficient of determination of 0.90.

### 3.1. Sample Database

The calibration set of 284 samples included 104 alfalfa and 180 grass samples. Each forage sample in the database had associated forage constituent values, including NDF, IVTD, NDFD, ADF, ADL, and CP (Table 1). The range and standard deviation of each parameter for the calibration and validation sets are detailed in Table 1. Combining the two species increased the range and standard deviation of the calibration dataset.

To understand the utility of this data to develop prediction models and compare the two instruments, the maximum R^2^MAX was calculated. The R^2^MAX can be computed from

(4) $$R^{2}\mathrm{MAX}=\frac{{\mathrm{SDL}}^{2}-{\mathrm{SEL}}^{2}}{{\mathrm{SDL}}^{2}}$$

where the SDL and SEL are the standard deviation and the standard error of the laboratory for the calibration dataset and a particular forage constituent. This value would be attainable if no error was introduced by the spectra or model [18]. The result of this analysis demonstrated the utility of these datasets to support calibration development with an R^2^MAX of 0.99, 1.0, 0.99, 0.98, 0.94, and 1.0, for NDF, IVTD, NDFD, ADF, ADL, and CP, respectively.

### 3.2. Relative Instrument Performance

The relative performance of the FOSS and NEO instruments was compared by developing calibration models that included both grass and alfalfa samples. Separate species calibrations were also considered, but the predictive performance was comparable, so, in effect, the mixed model summarizes the results. The best models based on simultaneously minimizing the SEC and the gap between SEC and SECV utilized a Savitzky–Golay derivative filter with a smoothing kernel of length 11, quadratic interpolation, and first derivative spectral transformations (Table 1).

The SEC for the constituent models ranged from 0.44 to 3.5, whereas the associated R^2^C ranged from 0.87 to 0.97 (Table 1). The model developed with the NEO had an average increase in SEC of 0.07 and a decrease in R^2^C of 0.01. The two instruments showed corresponding trends in model performance criteria SEC, SECV, R^2^C, and R^2^CV, wherein better statistics for a particular constituent yielded better performance results for both instruments.

The number of latent variables needed to explain 95% of the variance in X was the same between instruments. The number of latent variables used in these models (four) is lower than the 3–16 reported by Acosta et al. [9] in the split-dataset models, or the 5–13 reported by Berzaghi et al. [10]. Consequently, a cut-off of 99% explained X variance was also explored, improving calibration model performance in terms of SEC and R^2^C, but increasing the difference between the SEC and SECV, indicating overfitting.

**Figure 3:**
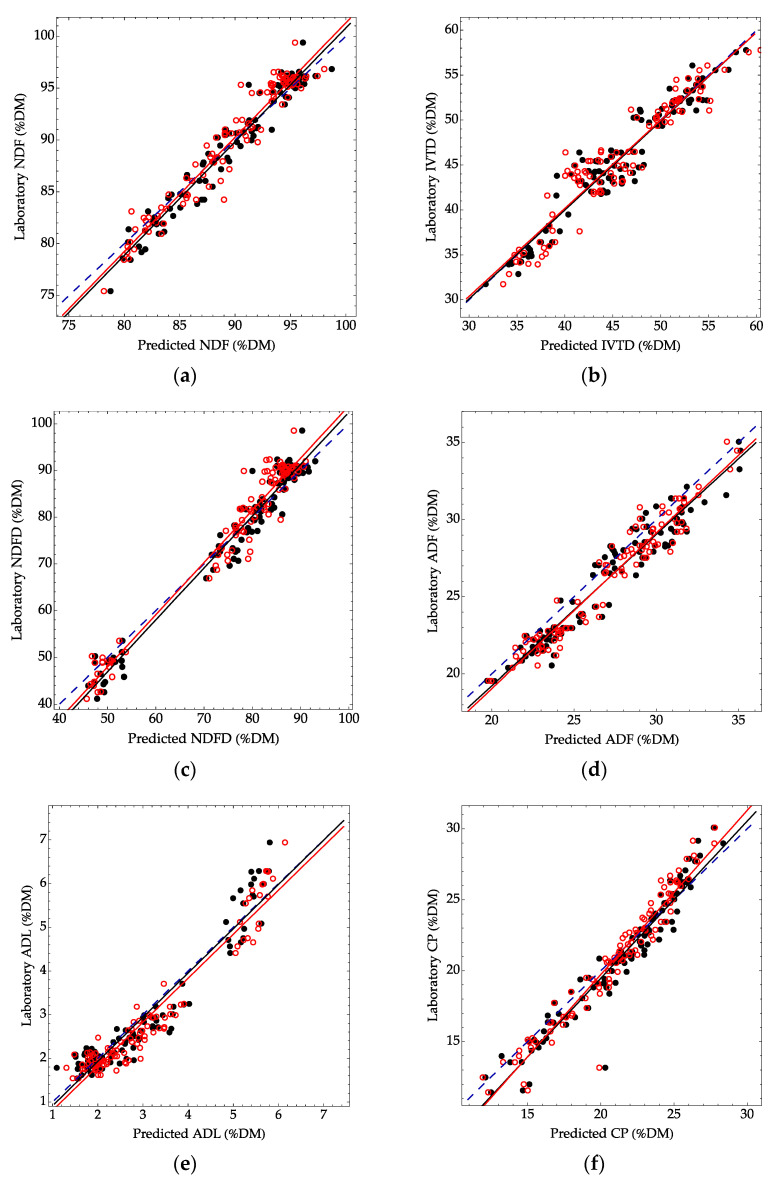
Validation scatter plots for an independent set of 20 alfalfa and 80 grass samples. Each point represents a laboratory result ((**a**) NDF, (**b**) IVTD, (**c**) NDFD, (**d**) ADF, (**e**) ADL, (**f**) CP) compared to a predicted result using the mixed-species calibration model for the FOSS (solid black circles) and NEO (open red circles). The dashed blue line represents a 1:1 agreement between laboratory and predicted values. The solid black and red lines correspond to a linear model between the laboratory and predicted values for the FOSS (black) and NEO (red).

The similarity between the two instruments was supported by using the developed models to predict an independent set of samples (Table 2, Figure 3). Here the R^2^P ranged from 0.90 to 0.97 for the FOSS, with an average difference of 0.002 between the FOSS and NEO instruments. The SEP for the constituents ranged from 0.38 to 3.8. The average difference in SEP between the NEO and FOSS instruments was 0.09.

These results agree with both Acosta et al. [9] and Berzaghi et al. [10], who tested both FOSS and low-cost spectrometers that employed digital micromirror device (DMD) MEMS technology. Although the technology is different from the NEO device studied here, the limitations of the device were greater in terms of wavelength resolution of the acquired spectra. At 16 nm, the NEO device has half the reported wavelength resolution of the DMD (8 nm) and one-eighth that of the FOSS (2 nm). However, the wavelength repeatability is likely to be higher than the resolution. At the time of writing, this information was not published for the NEO.

## 4. Conclusions

Forage nutritive value prediction models were developed using a dataset of near-infrared reflectance spectra and laboratory reference values for NDF, IVTD, NDFD, ADF, ADL, and CP, from dried and ground alfalfa and grass samples. The data and models developed in this study demonstrate the utility of low-resolution NIR spectra in this application, and the potential of new, low-cost spectrometers in the application of predicting forage nutritive value.

## Figures and Tables

**Table 1:** Statistics of calibration results for predicting forage nutritive value from a model that combines data from fresh alfalfa (N = 104) and fresh grass (N = 180) samples using the NIRSystems 6500 (FOSS), and the NeoSpectra-Scanner handheld instrument (NEO).

	NDF		IVTD		NDFD		ADF		ADL		CP	
N	284											
Units	%DM											
SEL	0.45		0.57		1.34		0.74		0.29		0.21	
Min.	76		21		40		18		1.5		13	
Mean	87		46		70		30		3.7		20	
Max.	97		64		93		38		7.2		33	
Stdev.	4.5		13		13		5.8		1.2		4.8	
	FOSS	NEO	FOSS	NEO	FOSS	NEO	FOSS	NEO	FOSS	NEO	FOSS	NEO
LVs	4	4	4	4	4	4	4	4	4	4	4	4
SEC	1.4	1.5	2.3	2.4	3.3	3.5	1.5	1.5	0.44	0.44	1.3	1.3
R^2^C	0.91	0.89	0.97	0.97	0.94	0.93	0.93	0.93	0.87	0.87	0.93	0.93
SECV	1.5	1.6	2.4	2.4	3.6	3.7	1.6	1.7	0.47	0.46	1.3	1.3
R^2^CV	0.91	0.88	0.97	0.96	0.94	0.93	0.93	0.92	0.87	0.87	0.93	0.93

N—number of samples, Stdev.—standard deviation of the sample set, SEL—standard error of the laboratory reference, NDF—neutral detergent fiber, IVTD—in vitro true digestibility, NDFD—neutral detergent fiber digestibility, ADF—acid detergent fiber, ADL—acid detergent lignin, CP—crude protein, % DM—percent dry matter, OR—outlier removal, LV—number of latent variables used in the model, SEC(V)—standard error of calibration (5-fold cross-validation), R²C(V)—coefficient of determination for calibration (cross-validation).

**Table 2:** Statistics of validation results for predicting forage nutritive value from a model that combines data from fresh alfalfa (N = 20) and fresh grass (N = 80) samples using the NIRSystems 6500 (FOSS), and the NeoSpectra-Scanner handheld instrument (NEO).

	NDF		IVTD		NDFD		ADF		ADL		CP	
N	100											
Units	%DM											
Min.	76		32		41		20		1.5		11	
Mean	87		46		77		26		2.9		21	
Max.	95		58		99		35		6.9		30	
Stdev.	4.2		6.5		16		3.8		1.4		4.4	
	FOSS	NEO	FOSS	NEO	FOSS	NEO	FOSS	NEO	FOSS	NEO	FOSS	NEO
R^2^P	0.95	0.94	0.92	0.90	0.97	0.96	0.93	0.94	0.91	0.93	0.93	0.93
RMSEP	1.4	1.6	1.8	2.1	3.3	3.8	1.4	1.3	0.42	0.42	1.3	1.3
SEP	1.4	1.5	1.8	2.1	3.3	3.7	1.0	0.96	0.42	0.38	1.2	1.3
Bias	−0.17	−0.22	−0.08	−0.07	−0.13	−0.96	−0.90	0.89	−0.06	0.17	−0.40	0.11
Slope	1.1	1.1	0.99	0.98	1.1	1.1	0.98	1.0	1.0	1.0	1.1	1.2

N—number of samples, Stdev.—standard deviation of the sample set, SEL—standard error of the laboratory reference, NDF—neutral detergent fiber, IVTD—in vitro true digestibility, NDFD—neutral detergent fiber digestibility, ADF—acid detergent fiber, ADL—acid detergent lignin, CP—crude protein, % DM—percent dry matter, RMSEP—root mean standard error of prediction, SEP—standard error of prediction, R²P—coefficient of determination.
